# Methylation-driven mechanisms of allergic rhinitis during pollen and non-pollen seasons using integrated bioinformatics analysis

**DOI:** 10.3389/fgene.2024.1242974

**Published:** 2024-04-18

**Authors:** Pengcheng Sun, Yi Wang, Xing Liu, Zhuqing Li, Diankun Cui, Qianru Li, Qi Wang, Ji Wang

**Affiliations:** ^1^ College of Traditional Chinese Medicine, Beijing University of Chinese Medicine, Beijing, China; ^2^ National Institute of Traditional Chinese Medicine Constitution and Preventive Medicine, Beijing University of Chinese Medicine, Beijing, China; ^3^ Qinghai Golmud Jianqiao Hospital, Golmud, Qinghai, China

**Keywords:** allergic rhinitis, asthma, pollen season, gene expression profile, DNA methylation profile, bioinformatics

## Abstract

**Background:**

Allergic rhinitis (AR) is a widespread allergic airway disease that results from a complex interplay between genetic and environmental factors and affects approximately 10%–40% of the global population. Pollen is a common allergen, and exposure to pollen can cause epigenetic changes. However, the mechanism underlying pollen-induced DNA methylation changes and their potential effects on the allergic march are still unclear. The purpose of this study was to explore the methylation-driven mechanisms of AR during the pollen and non-pollen seasons using bioinformatics analysis and to investigate their relationship with asthma.

**Methods:**

We downloaded DNA methylation and gene expression data from the GEO database (GSE50387: GSE50222, GSE50101) and identified differentially methylated positions (DMPs) and differentially expressed genes (DEGs) during the pollen and non-pollen seasons using the CHAMP and limma packages. Through correlation analysis, we identified methylation-driven genes and performed pathway enrichment analysis to annotate their functions. We incorporated external data on AR combined with asthma (GSE101720) for analysis to identify key CpGs that promote the transformation of AR to asthma. We also utilized external data on olive pollen allergy (GSE54522) for analysis to validate the methylation-driven genes. Weighted correlation network analysis (WGCNA) was employed to identify gene modules significantly correlated with pollen allergy. We extracted genes related to the key methylation-driven gene *ZNF667-AS1* from the significant module and performed pathway intelligent clustering using KOBAS-i. We also utilized gene set enrichment analysis to explore the potential function of *ZNF667-AS1*.

**Results:**

We identified 20 and 24 CpG-Gene pairings during the pollen and non-pollen seasons. After incorporating external data from GSE101720, we found that *ZNF667-AS1* is a key gene that may facilitate the transformation of AR into asthma during the pollen season. This finding was further validated in another external dataset, GSE54522, which is associated with pollen allergy. WGCNA identified 17 modules, among which the blue module showed significant correlation with allergies. *ZNF667-AS1* was located in the blue module. We performed pathway analysis on the genes correlated with *ZNF667-AS1* extracted from the blue module and identified a prominent cluster of pathways in the KOBAS-i results, including Toll-like receptor (*TLR*) family, *MyD88*, *MAPK*, and oxidative stress. Gene set enrichment analysis around *cg05508084* (paired with *ZNF667-AS1*) also indicated its potential involvement in initiating and modulating allergic inflammation from the perspective of *TLR* and *MAPK* signaling.

**Conclusion:**

We identified methylation-driven genes and their related pathways during the pollen and non-pollen seasons in patients with AR and identified key CpGs that promote the transformation of AR into asthma due to pollen exposure. This study provides new insights into the underlying molecular mechanisms of the transformation of AR to asthma.

## Introduction

Allergic diseases, including allergic rhinitis (AR), asthma, and atopic dermatitis, represent significant health concerns in global populations (Paller et al., 2019; Miller et al., 2021; Ständer, 2021; Zhang et al., 2021). There is a close association between allergic diseases, and the development of AR may be related to the manifestation of systemic allergy, including asthma (Bousquet et al., 2020b; Dharmage et al., 2022). Thirty percent of individuals with rhinitis also have asthma, and over eighty percent of individuals with asthma have symptoms of rhinitis (Nappi et al., 2022).

Pollen is a widespread aeroallergen that can induce allergic disease (Taylor et al., 2007; Schutzmeier et al., 2022). For now, it is recognized that more than 150 pollen allergens originating from grasses, trees, and weeds contribute significantly to an allergic response (Xie et al., 2019). Worldwide, the sensitization rate to pollen allergens is approximately 40% (Pointner et al., 2020). According to the International Study of Asthma and Allergies in Childhood (ISAAC), the prevalence of pollen sensitization in children increases by approximately 0.3% per year (Suanno et al., 2021). Approximately 20% of the US population suffers from pollen allergies, while about 20% of the population in Europe is affected by grass pollen allergies (García-Mozo, 2017). About 18.5% of the population in the northern grasslands of China is affected by pollen-induced AR (Wang et al., 2018). The incidence of pollen allergies exhibits geographic variation, influenced by bioclimatic conditions and the distribution of allergenic plants. Several studies have demonstrated that the exposure to pollen can induce DNA methylation (one of the epigenetic control of gene expression) for AR patients (North et al., 2018; Watanabe et al., 2021; Yang et al., 2022). The underlying reason is that environmental exposures can influence DNA methylation, which mediates the interaction between the environment and genotype to impact clinical phenotype (Law and Holland, 2019). Concerning the relationship between methylation and AR, epigenome-wide association studies (EWAS) have found specific CpG sites in AR patients (Qi et al., 2020). DNA methylation may help distinguish allergic patients from healthy individuals (Choi et al., 2021). According to the sensitization on cyclic pollens or year-round allergens, AR can be classified as seasonal or perennial (Greiner et al., 2011). For seasonal allergic rhinitis (SAR) patients, a previous study demonstrated that DNA methylation profiles instead of gene expression profiles can clearly and robustly distinguish them from healthy controls, during the pollen and non-pollen seasons (Nestor et al., 2014). However, how pollen-induced DNA methylation affects SAR patients by changing gene expression has not been fully elucidated.

**FIGURE 1:**
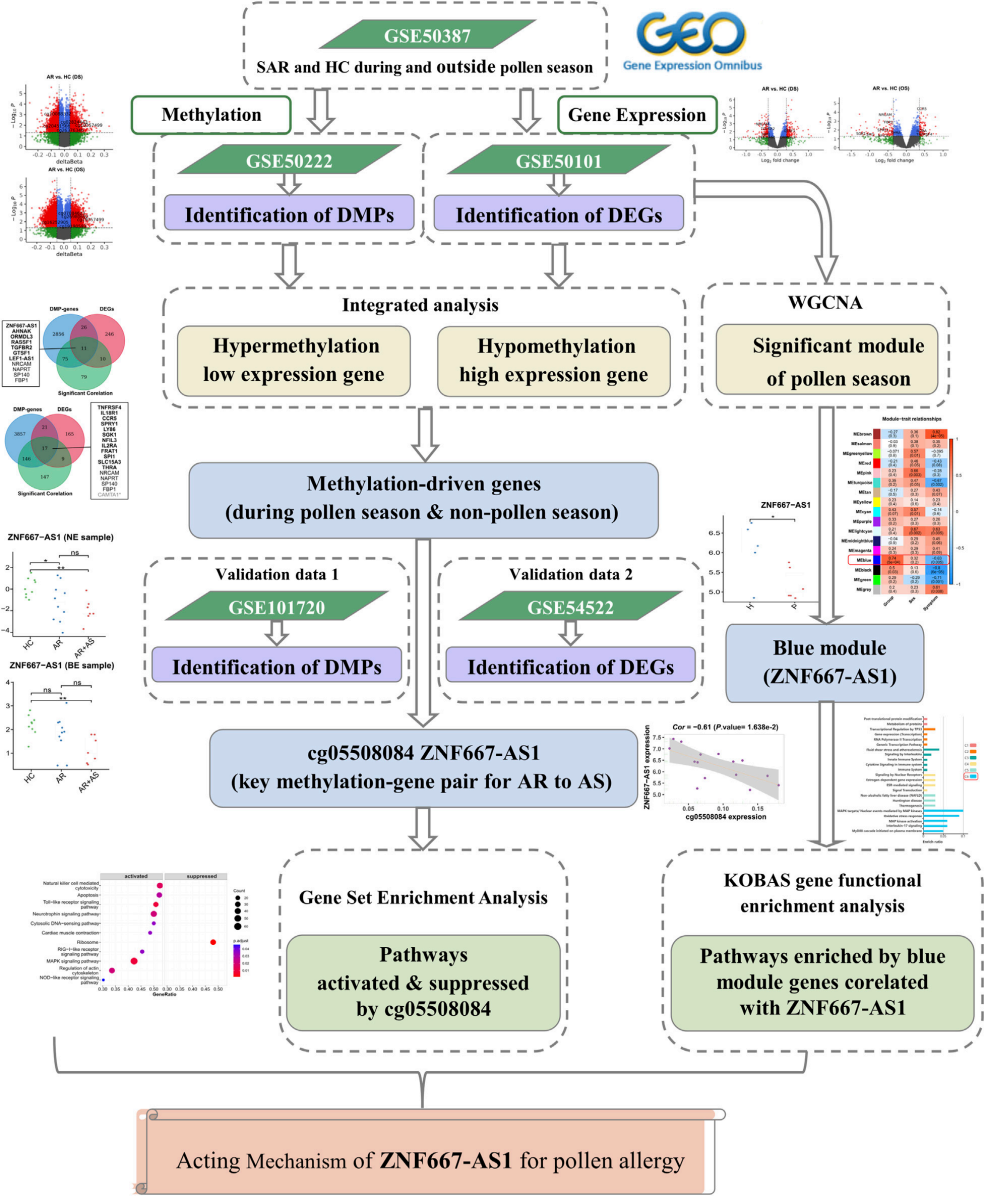
The flowchart of the analysis. DMP: differentially methylated position; DEG: differentially expressed gene; WGCNA: weighted correlation network analysis; AR: allergic rhinitis; SAR: seasonal allergic rhinitis; AS: asthma; HC: healthy control.

To investigate the role of DNA methylation in the regulation of gene expression in SAR, we downloaded publicly available data and performed a correlation analysis of significant differentially methylated positions (DMPs) with critical differentially expressed genes (DEGs). Our bioinformatics analysis aimed to identify potential epigenetic mechanisms that contribute to the development and progression of SAR. To support our findings and investigate the role pollen plays in the evolution of SAR to asthma, we incorporated external data on the co-morbidity of AR and asthma and compared it with the analysis results of SAR during the pollen and non-pollen seasons. The flowchart of the analysis is shown in Figure 1.

## Materials and methods

### DNA methylation and gene expression data resources

The microarray datasets GSE50222 (Last update date: 22 Mar 2019) and GSE50101 (Last update date: 13 Aug 2018) were downloaded from the GEO database (http://www.ncbi.nlm.nih.gov/geo/) (Edgar et al., 2002; Barrett et al., 2013). They were derived from a super series GSE50387 (Nestor et al., 2014) that contains methylation chip and gene chip data of CD4^+^ T cells from the same group of AR patients who were recruited from Sweden people in Europe. Positive skin prick test results and/or positive ImmunoCAP Rapid results to birch and/or grass pollen were the two main indicators of SAR, which were both negative in health controls. Patients with perennial symptoms or asthma were not included. GSE50222 is a methylation dataset that contains 32 samples, which are divided into four groups: AR of the pollen season (n = 8), AR of the non-pollen season (n = 8), healthy controls (HCs) of the pollen season (n = 8), and HCs of the non-pollen season (n = 8). The chip platform used was GPL13534 (Illumina HumanMethylation450 BeadChip). GSE50101 is a gene expression dataset containing 38 samples, which are divided into four groups: AR of the pollen season (n = 9), AR of the non-pollen season (n = 9), HCs of the pollen season (n = 10), and HCs of the non-pollen season (n = 10). The chip platform used was GPL10558 (Illumina HumanHT-12 V4.0 Expression Beadchip).

### Data preprocessing and DMP screening

R (version 4.2.3) (R Core Team, 2023) was used for data preprocessing. First, we determined the missing rates for both the probes and the samples, and we kept only the probes and samples with missing rates under 10%. We eliminated ‘bad’ probes like cross-reactive/non-specific probes and BOWTIE2 multi-mapped probes (Chen et al., 2013; Pidsley et al., 2016). Afterward, the beta matrix was calculated using the following equation: β = M/(M + U+100). For our samples, we extracted the grouping information using the GEOquery package (Davis and Meltzer, 2007). To further filter out the probes, we used the ChAMP package (version 2.24.0) (Morris et al., 2014; Tian et al., 2017) and its champ.filter function (Wang et al., 2023).

We conducted a differentially methylated positions (DMP) analysis between the AR group and the HC group, both during the pollen and non-pollen seasons. Taking into account the effect of Type I and Type II probes, we first conducted quality control using champ.QC function and then applied the champ.norm function for standardization. Additionally, we utilized champ.SVD function to look at the principal components and champ.DMP to find DMPs.

Firstly, we conducted quality control using champ.QC function and then applied champ.norm function for standardization to correct for the effects of Type-I and Type-II probes. We also used champ.SVD function to examine principal components and identified DMPs using champ.DMP function. The following criteria were set to further screen for methylation-driven sites (Guo et al., 2020):

(1) $\text{abs}\left(\Delta \beta \right)\;>\text{ mean}\left(\text{abs}\left(\Delta \beta \right)\right)+2*\text{sd}\left(\text{abs}\left(\Delta \beta \right)\right)$

(2) *p*-value < 0.05
(3) The location belongs to the promoter regions (1stExon, 5′UTR, TSS1500, TSS200).

### Data preprocessing and DEG screening

We conducted a DEG analysis between the AR group and the HC group, both during the pollen and non-pollen seasons.

First, we determined the missing rate for both the samples and the probes, keeping only the probes with a missing rate of less than 10%. According to detection *p*-values, we performed background correction on the samples using the nec function in the limma package (version 3.50.3) (Ritchie et al., 2015). The second step involved probe filtration. The raw gene expression matrix was split by group. Significant probes were defined as those with *p*-value < 0.05 in at least 50% of the samples. For non-significant probes, their expression values were replaced with the average expression value of these probes across all samples within each group. Third, we used limma package to do DEG analysis with filtering criteria set to abs(log_2_FC) > mean(abs(log_2_FC)) + 2*sd(abs(log_2_FC)) and *p*-value < 0.05. Finally, the probes were annotated and the duplicate genes were removed.

We create a Venn diagram with the R package VennDiagram (v1.73) (Chen, 2018) to depict the link between the upregulated and downregulated DEGs discovered during the pollen and non-pollen seasons. Then, we selected the unique parts of these four groups of genes and conducted gene function enrichment analysis for each group with Kyoto Encyclopedia of Genes and Genomes database (KEGG) (Kanehisa and Goto, 2000; Kanehisa et al., 2021) using clusterProfiler package (version 4.7.1.003) (Yu et al., 2012; Wu et al., 2021) and org.Hs.eg.db package (version 3.16.0) (Carlson, 2021).

### Integrated analysis of DNA methylation and gene expression data

We matched the samples between methylation and gene chips according to the patient ID, and obtained the correspondence between CpG sites and genes through annotation information. Then we calculated the Spearman correlation coefficient between the methylation β values at CpG sites and the gene expression values. CpG-Gene pairs were filtered with *p*-value < 0.05 and *Cor* < −0.4. Only the DMP-DEG pairs were retained. Finally, we compared the results of DMP related genes, DEGs, and the genes with significant correlation coefficients using a Venn diagram both in the pollen season and the non-pollen season.

We conducted gene function enrichment analysis on the methylation-driven genes obtained during the pollen and non-pollen seasons based on the hypergeometric distribution. The pathway databases used included KEGG database (Kanehisa and Goto, 2000; Kanehisa et al., 2021), Reactome database (Gillespie et al., 2022), and Gene Ontology (GO) database (Ashburner et al., 2000; Gene Ontology Consortium, 2021) limited to biological processes (BP). Enrichment analysis was performed using the enrichKEGG function (Yu et al., 2012; Wu et al., 2021), enrichPathway function (Yu and He, 2016), and enrichGO function (Yu et al., 2012; Wu et al., 2021) for KEGG, Reactome, and GO, respectively. Finally, the results were compared and visualized.

### Comparison of methylation-driven genes with data on allergic comorbidity

The allergic comorbidity sequencing dataset GSE101720 (Last update date: 17 Dec 2020) was also downloaded from the GEO database. It includes samples from people who have AR and asthma, people who merely have AR and healthy people (Giovannini-Chami et al., 2018). The dataset contains nasal epithelial samples (n = 26, AR with asthma = 7, AR = 10, HC = 9) and bronchial epithelial samples (n = 26, AR with asthma = 7, AR = 10, HC = 9) that were sequenced on the Illumina NextSeq 500 platform (GPL18573).

With samples from the nasal epithelium and bronchial epithelium, we used the limma package to identify DEGs between the AR with asthma group and the HC group, as well as between the AR group and the HC group. The filtering criterion (Luo et al., 2019) for DEGs was set as *p*-value < 0.05 and abs(log_2_FC) > (mean(abs(log_2_FC)) + 2*sd(abs(log_2_FC))).

We retrieved the methylation-driven genes from the previous section and compared them to the DEGs from this part.

### Comparison of methylation-driven genes with data on olive pollen allergy

The data on olive pollen allergy, GSE54522 (Last update date: 25 Mar 2019), was downloaded from the GEO database. This dataset includes peripheral blood mononuclear cells (PBMCs) from 6 olive pollen-allergic patients and 6 HC subjects, which were stimulated with olive pollen for 24 h (Calzada et al., 2015). The olive pollen-allergic patients met the following criteria: seasonal rhinitis and/or asthma from April to June, a positive skin prick test result for *O. europaea* pollen extract, and no previous immunotherapy. From this dataset, we selected samples stimulated with pollen extract during the pollen season (n = 6) and compared them to the stimulated samples from the HC group (n = 5). The filtering criterion (Luo et al., 2019) for identifying DEGs was set as a *p*-value < 0.05 and abs(log_2_FC) > mean(abs(log_2_FC)) + 2*sd(abs(log_2_FC)). We then compared the methylation-driven genes with the DEGs identified in this analysis.

### Weighted correlation network analysis for GSE50101

We utilized Weighted Correlation Network Analysis (WGCNA) (Langfelder and Horvath, 2008) to analyze the gene expression data from GSE50101, aiming to explore the gene expression patterns in samples during the pollen season. To ensure data heterogeneity and analysis accuracy, the following gene filtering steps were applied: a) Genes were tested using the goodGenes function; b) Genes with an upper 25% median absolute deviation were selected (Wei et al., 2020). For sample selection in WGCNA, the following steps were performed: a) Samples were tested using the goodSamples function; b) A sample clustering plot was generated using the hclust function, and outlier samples were removed. The prepared gene expression matrix was used to calculate the Soft Threshold (with *R* ^2^ cutoff set to the default value of 0.85). Considering the involvement of numerous genes in allergies, the minModuleSize was set to 100 to construct a weighted gene co-expression network. For the obtained gene modules, module eigengenes were calculated and used to assess the correlation with traits (grouping, gender, symptom scores). Key modules were selected based on a correlation threshold of *Cor* > 0.4 and a *p*-value < 0.05. Within the key module, the correlation between all genes and *ZNF667-AS1* expression was calculated, and genes with a correlation of *Cor* > 0.4 and a *p*-value < 0.05 were selected. Pathway analysis was performed using KOBAS-i (Bu et al., 2021), with pathway databases including KEGG, Reactome, BioCyc, and PANTHER. Pathways with a Corrected *p*-value < 0.05 were further subjected to intelligent clustering and visualization.

### Gene set enrichment analysis for *cg05508084*

*ZNF667-AS1* was identified as a key methylation-driven gene during the pollen season, and it is regulated by *cg05508084*. To explore the potential function of *ZNF667-AS1*, we calculate the association between *cg05508084* and the levels of gene expression for the pollen season. Following the results of the correlation analysis, single-gene Gene Set Enrichment Analysis (GSEA) was conducted with the databases KEGG and Reactome from MSigDB (Subramanian et al., 2005) using the msigdbr package (version 7.5.1) (Dolgalev, 2022). Visualization was done of the pathways that *cg05508084* either activated or inhibited.

## Results

### Identification of DMPs during the pollen and non-pollen seasons

**FIGURE 2:**
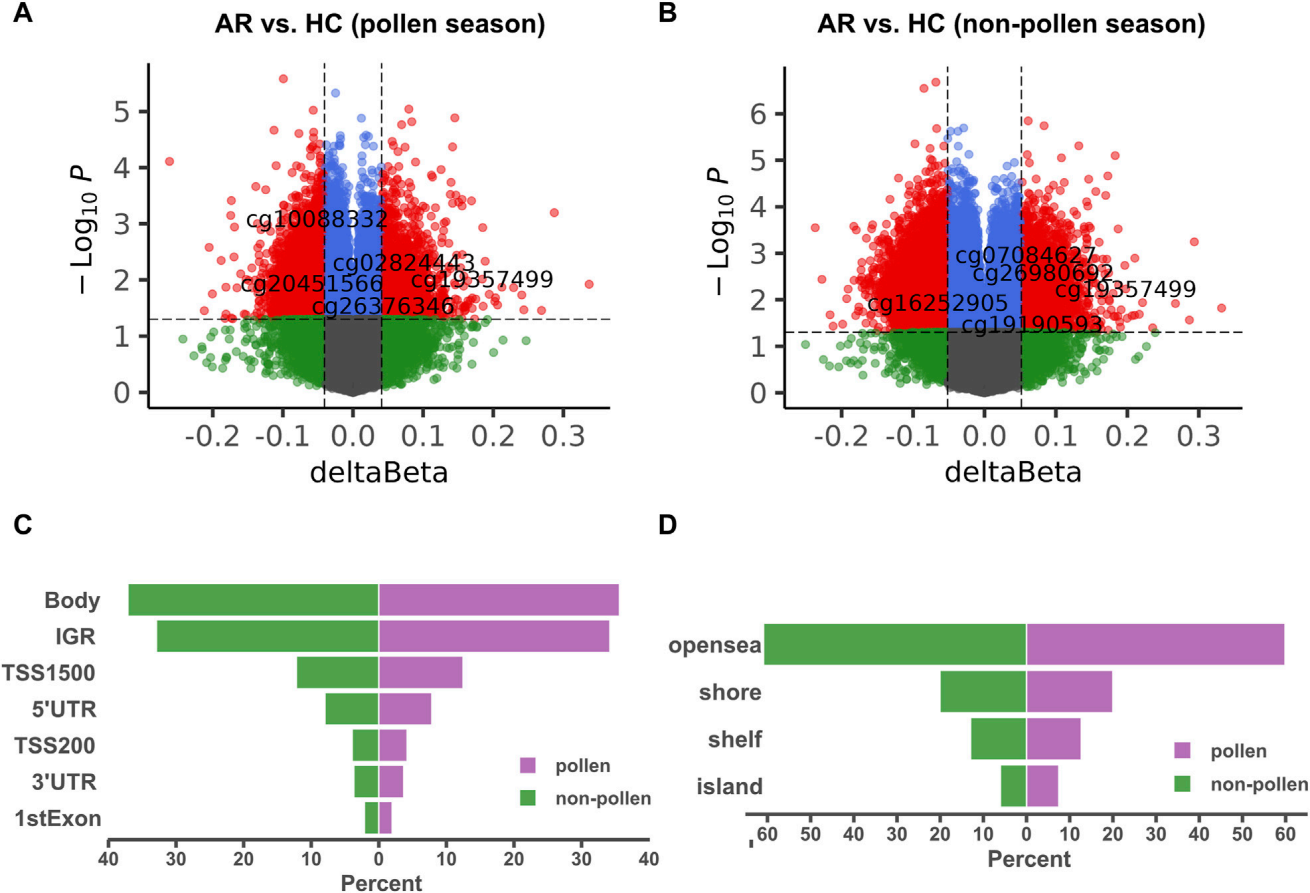
Identification of DMPs between AR and HC groups in GSE50222. **(A, B)** The volcano map of the DMPs during the pollen and non-pollen seasons. The X-axis corresponds to the β change value, while the Y-axis corresponds to the -log_10_(*p*-value). The red nodes show the hypomethylation and hypermethylation DMPs. **(C, D)** The distribution of the DMPs during the pollen and non-pollen seasons. The comparison was performed on exact promoter region and CpG island separately. DMP: differentially methylated position; AR: allergic rhinitis; HC: healthy control.

The general workflow for analyzing Illumina HumanMethylation450 BeadChip data includes quality control, normalization, differential expression analysis, and annotation. Our quality control consists of three steps: a) filtering based on probe and sample missing rate, removing probes and samples with missing rates exceeding 10%; b) filtering based on unique probe annotation, removing ‘bad’ probes with non-unique annotations; c) filtering based on Methylated matrix, UnMethylated matrix, and Detected *p*-value. The study began by filtering out methylation probes that had a missing rate of over 10%. This resulted in 4,49,506 probes remaining out of the initial 4,85,577. Next, non-specific probes were filtered out, leaving 4,13,172 methylation probes. The probes were further filtered using champ.filter, with 3,89,332 remaining in the end. The beta matrix was standardized between type I and type II probes using champ.norm. DMP analysis was performed using champ.DMP, resulting in the identification of 11,275 DMPs during pollen season and 16,975 DMPs during the non-pollen season. In champ.DMP results, DMPs are annotated with genomic locations and corresponding genes. Of these, 2,987 DMPs of the pollen season and 4,458 DMPs of the non-pollen season were located in the promoter region. Volcano plots for DMPs are displayed in Figures 2A, B, while Figures 2C, D compare the distribution of DMPs of the pollen and the non-pollen seasons. The principal component analysis (PCA) plot and heatmap of the methylation data (GSE50222) are shown in Supplementary Figure S1.

### Identification of DEGs during the pollen and non-pollen seasons

**FIGURE 3:**
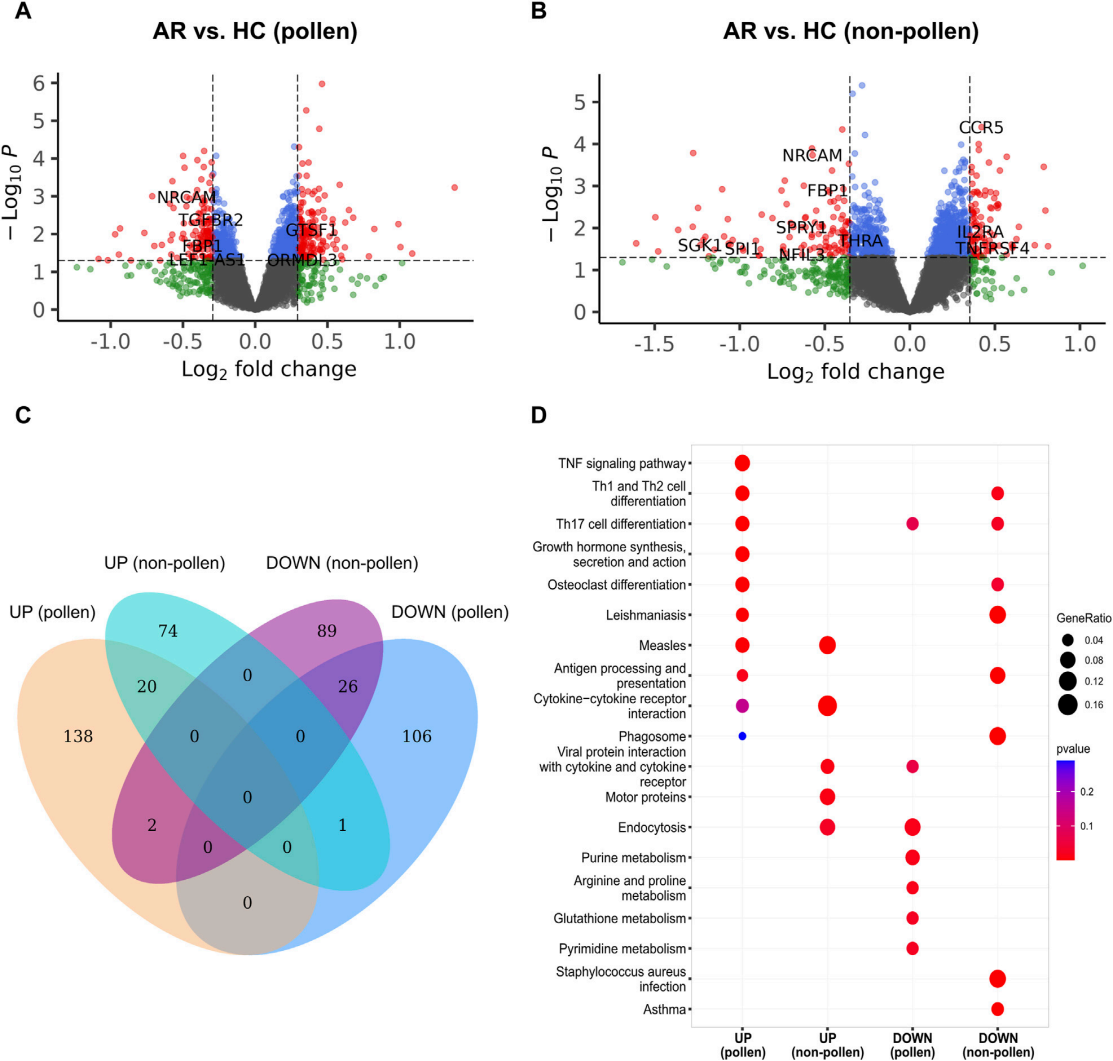
Identification of DEGs between AR and HC groups in GSE50101. **(A, B)** The volcano map of the DEGs during the pollen and non-pollen seasons. The X-axis corresponds to the log_2_FC, while the Y-axis corresponds to the -log_10_(*p*-value). The red node indicates the upregulated and downregulated DEGs. **(C)** The Venn plot of upregulated and downregulated DEGs during the pollen and non-pollen seasons. UP (pollen) and DOWN (pollen) represent the upregulated and downregulated DEGs of the pollen season. UP (non-pollen) and DOWN (non-pollen) represent the upregulated and downregulated DEGs of the non-pollen season. **(D)** KEGG pathways enriched by the subset of DEGs including UP (pollen), UP (non-pollen), DOWN (pollen), and DOWN (non-pollen). DEG: differentially expressed gene; AR: allergic rhinitis; HC: healthy control.

The analysis began by filtering out gene probes with a missing rate of over 10%, which resulted in 47,314 probes remaining out of the initial 47,323. In the AR group, 18,327 and 17,848 significant probes were retained during the pollen and non-pollen seasons. In the HC group, 18,591 and 18,371 significant probes were retained during the pollen and non-pollen seasons. Using log_2_FC and *p*-value criteria, a total of 293 DEGs were selected for the pollen season, out of which 160 were upregulated, and 133 were downregulated in AR. Similarly, 212 DEGs were selected for the non-pollen season, among which 95 were upregulated, and 117 were downregulated in AR. The volcano plots are shown in Figure 3A, B. The comparison of upregulated and downregulated DEGs during the pollen and non-pollen seasons can be seen in Figure 3C, D, the KEGG pathways enriched by the distinct genes in each of the four gene sets are contrasted. The PCA plot and heatmap of the gene expression data (GSE50101) are shown in Supplementary Figure S2.

### Results of integrated analysis of DNA methylation and gene expression data

**FIGURE 4:**
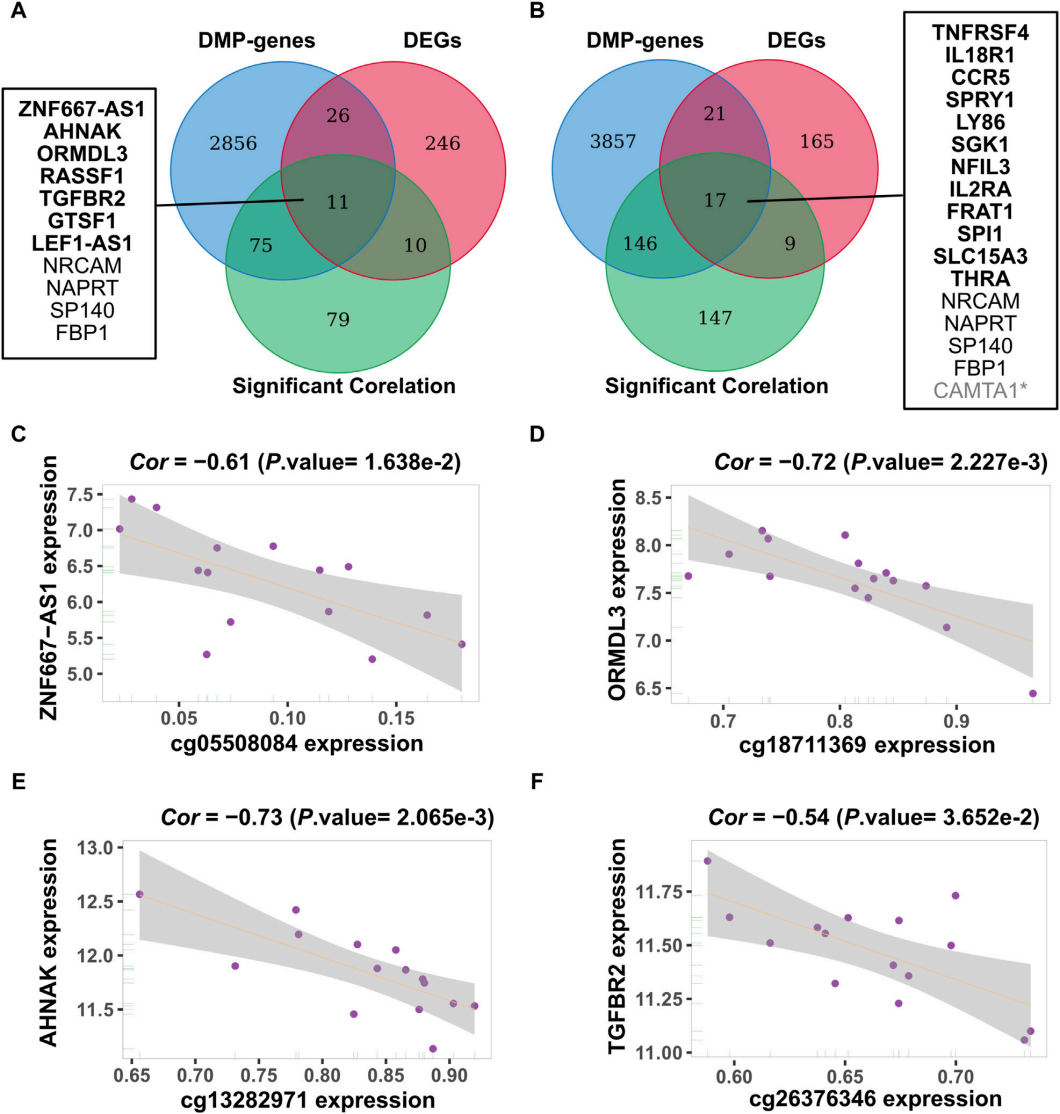
Identification of methylation-driven genes. **(A, B)** Identification of methylation-driven genes during the pollen and non-pollen seasons. The ellipses in three colors represent the DMP related gene set, the DEG gene set, and the gene set that exhibits a strong association between changes in methylation and expression. The middle part refers to the intersection of the three groups of data. The genes with the bolded name are specific methylation-driven genes. **(C–F)** Four specific methylation-driven genes of pollen season with correlation coefficient. The methylation-expression relationship was assessed using Spearman’s correlation coefficient, with methylation and expression levels plotted on the X-axis and Y-axis, respectively. DMP: differentially methylated position; DEG: differentially expressed gene. * Due to the fact that the methylated probes negatively correlated with the expression of *CAMTA1* are not differentially expressed, *CAMTA1* was ultimately excluded from the list of methylation-driven genes.

We identified 15 relevant pairs of samples by matching the sample IDs from the methylation (GSE50222) and gene expression data (GSE50101) during the pollen and non-pollen seasons. Subsequently, we determined the correlation coefficient between CpG sites and genes using consistent samples. A total of 262 and 470 CpG-Gene pairings were found during the pollen and non-pollen seasons when the filtering criterion was set as *p*-value < 0.05 and *Cor* < −0.4. By limiting CpG sites to DMPs and genes to DEGs, we identified 20 and 24 methylation-driven gene pairings during the pollen and non-pollen seasons. These CpG-Gene pairs include two types: hypermethylation low expression genes and hypomethylation high expression genes, as it is generally believed that methylation regulates transcription in a negative manner (Supplementary Tables S1, S2). The analysis revealed 11 unique genes associated with the pollen season and 16 unique genes associated with the non-pollen season, with four genes intersecting between them. The results of methylation-driven genes are shown in Figure 4A, B. The correlations of the four CpG-Gene pairs are shown in Figure 4C–F as examples. The expression level of these genes can be found in Supplementary Tables S3, S4.

### Results of gene functional enrichment analysis

Supplementary Figure S3A–C depict the results of the gene function enrichment analysis of methylation-driven genes during the pollen season. Detailed information can be obtained in Supplementary Tables S5–S7. They were primarily enriched in the *Hippo* signaling pathway (hsa04390), Neutrophil degranulation (R-HSA-6798695), cell growth (GO:0016049), etc.

Supplementary Figure S3D–F show the results of the gene function enrichment analysis performed on methylation-driven genes during the non-pollen season. Detailed information can be obtained in Supplementary Tables S8–S10. They were primarily enriched in Cytokine-cytokine receptor interaction (hsa04060), Viral protein interaction with cytokine and cytokine receptor (hsa04061), Signaling by Interleukin (R-HSA-449147), cytokine-mediated signaling pathway (GO:0019221), etc.

### Comparison results of methylation-driven genes with data on allergic comorbidity

**FIGURE 5:**
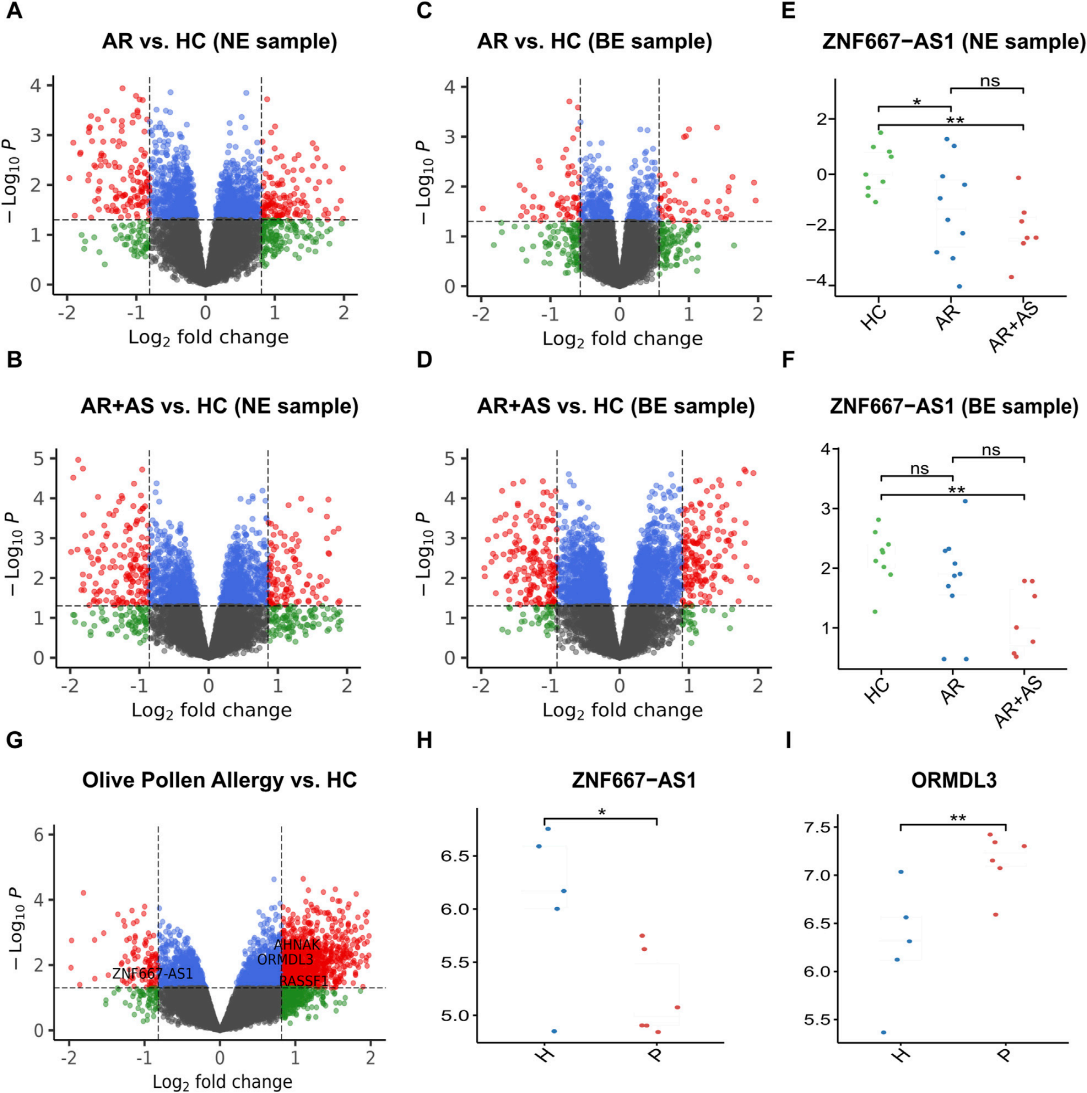
Result plot of validation using GSE101720 and GSE54522. **(A)** Volcano plot of AR vs. HC in GSE101720 (NE sample). **(B)** Volcano plot of AR with asthma vs. HC in GSE101720 (NE sample). **(C)** Volcano plot of AR vs. HC in GSE101720 (BE sample). **(D)** Volcano plot of AR with asthma vs. HC in GSE101720 (BE sample). **(E)** Expression level of *ZNF667-AS1* in GSE101720 (NE sample). **(F)** Expression level of *ZNF667-AS1* in GSE101720 (BE sample). **(G)** Volcano plot of olive pollen allergy patients vs. HC in GSE54522 (PBMC). **(H)** Expression level of *ZNF667-AS1* in GSE54522 (PBMC). **(I)** Expression level of *ORMDL3* in GSE54522 (PBMC). AR: allergic rhinitis; AS: asthma; HC: healthy control; NE: nasal epithelial; BE: bronchial epithelial.

The study analyzed the gene expression matrix of GSE101720, which includes 16,084 genes. Using this dataset, we investigated the DEGs in bronchial and nasal epithelial samples when comparing three groups: AR + asthma, AR, and HC. In bronchial epithelial samples, a total of 141 DEGs were identified, with 78 upregulated genes and 63 downregulated genes observed in the AR group compared to the HC group. When comparing the AR + asthma group to the HC group, 421 DEGs were found in bronchial epithelial samples, including 220 upregulated genes and 201 downregulated genes. Similarly, in nasal epithelial samples, comparison of the AR group with the HC group revealed 313 DEGs, with 162 upregulated genes and 151 downregulated genes. Upon comparing the AR + asthma group with the HC group, we identified 314 DEGs consisting of 145 upregulated genes and 169 downregulated genes in nasal epithelial samples. Volcano plots that illustrate the DEGs analysis results are presented in Figure 5A–D.

Our previous results revealed that *ZNF667-AS1* was a methylation-driven gene unique to the pollen season. It was found downregulated in both AR and AR + asthma patients in nasal epithelial samples and downregulated in AR + asthma patients in bronchial epithelial samples. The inter-group expression levels of the *ZNF667-AS1* gene in GSE101720 are presented in Figure 5E, F. Additionally, *NRCAM* was found to be a methylation-driven gene shared by pollen and non-pollen periods. It was downregulated in both AR and AR + asthma patients in nasal epithelial samples.

### Comparison results of methylation-driven genes with data on olive pollen allergy

The study analyzed the gene expression matrix of GSE54522, which includes 54,675 probes. Using this dataset, we investigated the DEGs in pollen-allergic patients and HC samples after olive pollen stimulation. A total of 1,321 DEGs were identified, with 1,206 upregulated genes and 115 downregulated genes observed in the allergic group compared to the HC group. We observed that four genes from the methylation-driven genes during the pollen season were also present among these DEGs. *ZNF667-AS1* was downregulated, while *AHNAK*, *ORMDL3*, and *RASSF1* were upregulated. Similarly, among the methylation-driven genes during the non-pollen season, two genes, *SLC15A3* and *FRAT1*, were also identified as upregulated genes.

### Results of weighted correlation network analysis for GSE50101

**FIGURE 6:**
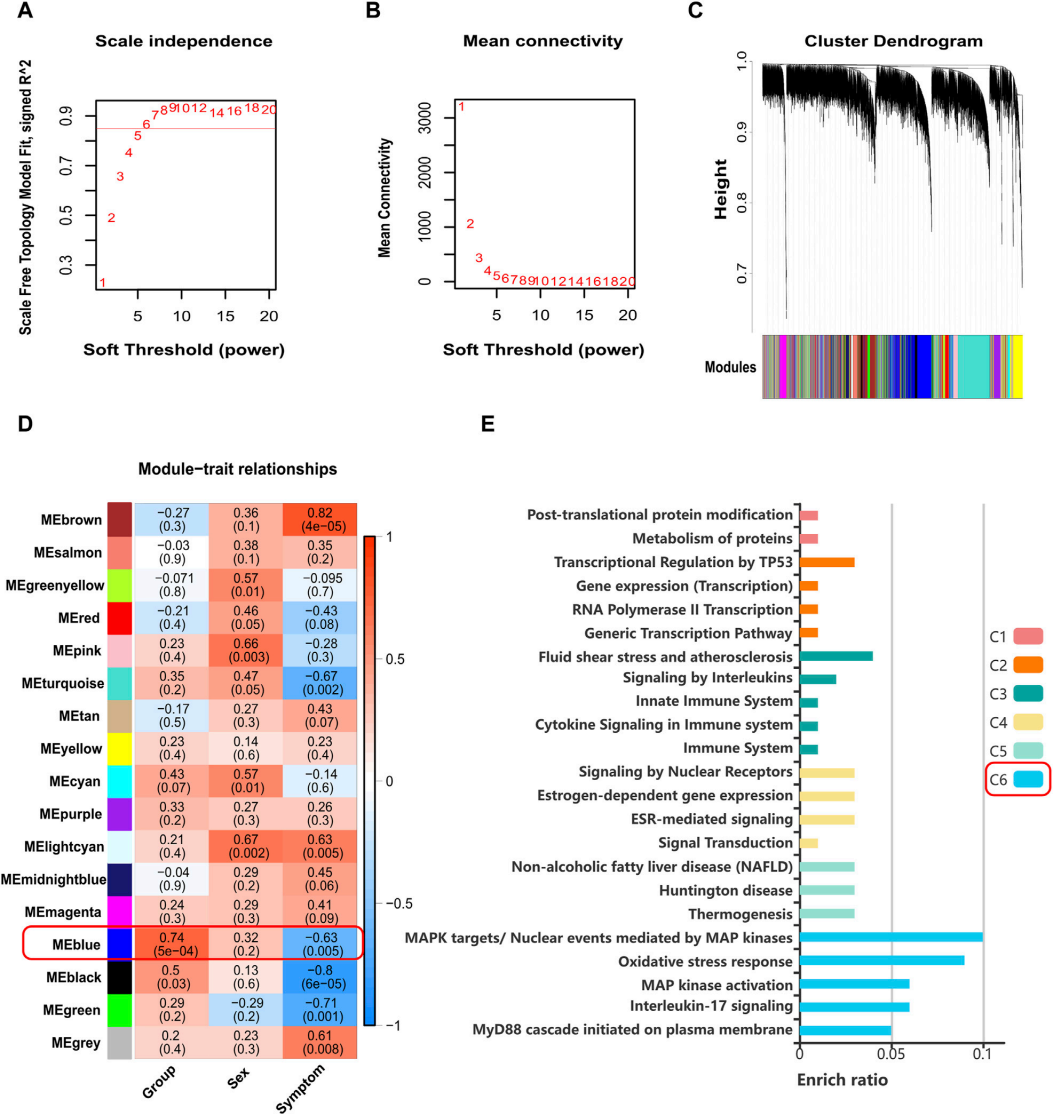
Results plot of WGCNA. **(A)** Scale independence and soft-threshold power. **(B)** Mean connectivity and soft-threshold power. **(C)** Cluster dendrogram of co-expressed genes. **(D)** Relationships of modules to traits. **(E)** Histogram of KOBAS-i pathway intelligent clustering for genes associated with *ZNF667-AS1* in the blue module. WGCNA: weighted correlation network analysis.

The pollen season expression matrix of GSE50101 contains a total of 47,314 genes and 19 samples. After probe annotation and removal of duplicates based on the highest expression value, 19,645 unique genes were remaining. Following filtering using the goodGenes function and Median Absolute Deviation, a total of 14,733 genes were included in the analysis. After filtering based on sample clustering and the goodSamples function, 18 samples were included in the analysis (Supplementary Figure S4A). The pickSoftThreshold function determined a soft threshold of 6, with *R* ^2^ cutoff of 0.85 (Figure 6A, B). The network construction resulted in 17 color modules (Figure 6C). Upon calculating the correlation between modules and traits, it was found that only the blue module showed significant correlation with grouping (*Cor* = 0.74, *p*-value < 0.05) (Figure 6D). The blue module consisted of 2,611 genes, and interestingly, *ZNF667-AS1* was also present in the blue module.

As WGCNA is a biological application used to identify highly correlated gene clusters (modules), and genes within these modules are presumed to exhibit coordinated interactions, we believe that the function of *ZNF667-AS1* can be investigated through studying the genes with which it exhibits co-expression within the blue module. The expression correlation between all genes in the blue module and *ZNF667-AS1* was calculated, resulting in 194 genes that showed significant correlation (*Cor* > 0.4, *p*-value < 0.05) (Supplementary Table S11). Pathway analysis using KOBAS-i was performed on these 195 genes (including *ZNF667-AS1*), and after filtering based on a Corrected *p*-value < 0.05, 7 pathways were enriched in KEGG, 41 pathways in Reactome, 1 pathway in PANTHER, while no pathways were enriched in BioCyc. Intelligent clustering of the pathways revealed 6 main clusters, with Cluster C6 being the largest, consisting of 18 pathways. This cluster includes pathways such as Toll-like receptor (*TLR*) family, *Myd88*, *MAPK*, oxidative stress, and others, as depicted in Figure 6E and Supplementary Table S12.

### Results of gene set enrichment analysis for *cg05508084*

**FIGURE 7:**
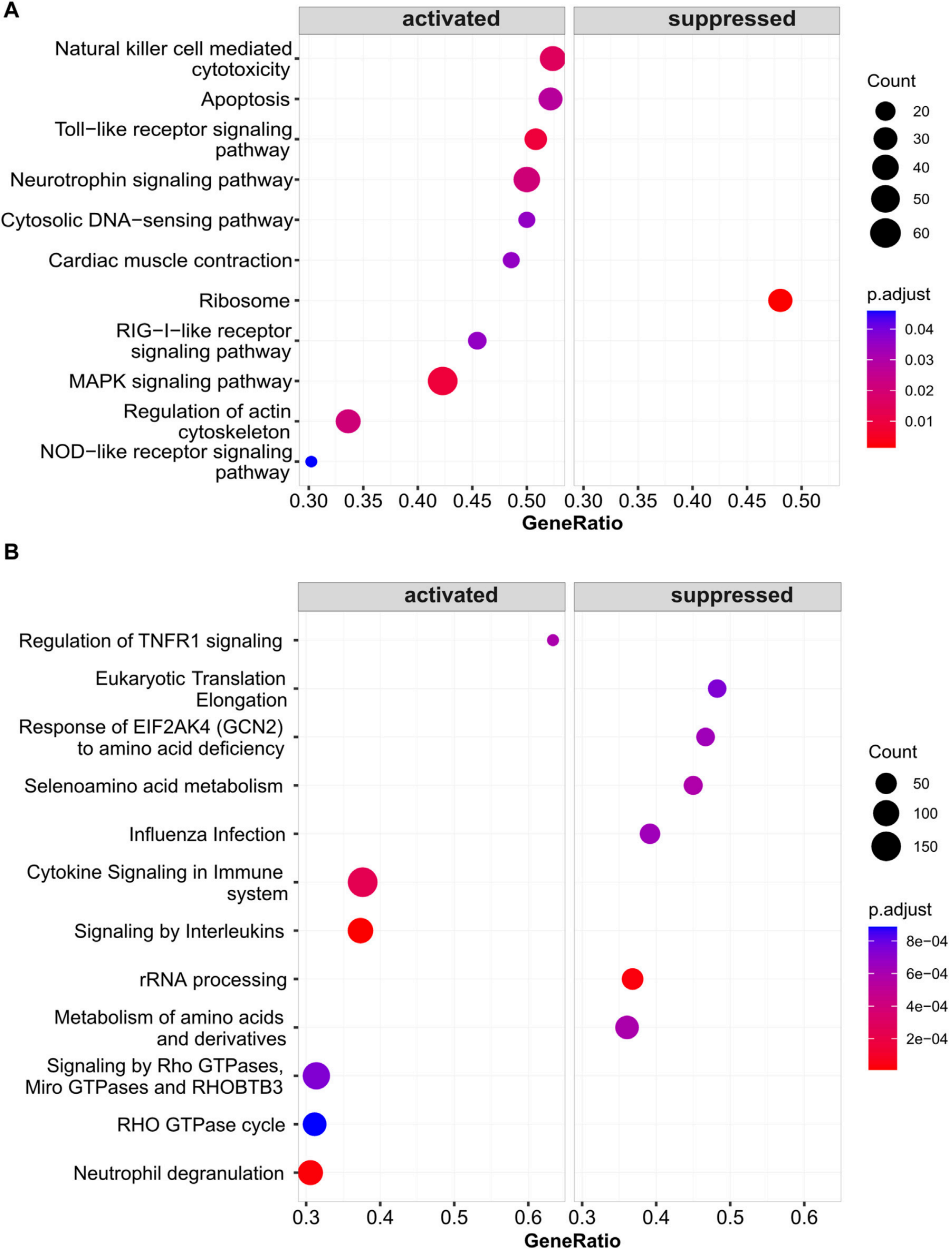
Results plot of Gene Set Enrichment Analysis for *cg05508084* (paired with *ZNF667-AS1*). **(A)** The KEGG pathways that are activated and inhibited by *cg05508084*. **(B)** The Reactome pathways that are activated and inhibited by *cg05508084*.

The corresponding CpG site for *ZNF667-AS1* is *cg05508084*. GSEA analysis revealed that the functional significance of the *cg05508084* site may be associated with the activation of *TLR* receptor signaling pathway and *MAPK* signaling pathway. The results are shown in Figure 7A,B.

## Discussion

Allergic rhinitis is a prevalent allergic airway disease characterized by symptoms such as sneezing, nasal congestion, nasal itching and rhinorrhea caused by immunoglobulin E (IgE)-mediated responses to inhaled allergens. These immune responses involve mucosal inflammation driven by type 2 cells, a subset of immune cells that produce cytokines responsible for inducing inflammation in response to allergens. Research suggests that exposure to allergens could trigger epigenetic changes (Cheng et al., 2014; Choi et al., 2021), leading to AR development with a susceptible genetic background (Bousquet et al., 2020a). Methylation has been shown to differentiate AR from the healthy individuals and to be associated with the development and exacerbation of AR (Li et al., 2016; Choi et al., 2021).

Pollen is one of the frequently occurring environmental allergens for AR (Skoner, 2001; Meng et al., 2020). Studies found that pollen-induced DNA methylation changes were correlated with symptom scores in AR patients, highlighting the role of epigenetic mechanisms in AR pathogenesis (Nestor et al., 2014; North et al., 2018; Watanabe et al., 2021; Tameeris et al., 2023). However, the specific effects of pollen season on AR patients have not been well elucidated. A bioinformatics study identified some specific signaling pathways for pollen season by comparing DMPs during the pollen and non-pollen seasons among AR patients and HCs (Yang et al., 2022). Our study goes further by integrating methylation profiling and gene expression profiling in a multi-omics approach, to identify methylation-driven genes specifically during the pollen and non-pollen seasons in AR to explore the unique epigenetic mechanisms of pollen season. This multi-omics approach of studying methylation-driven genes has been widely used in the study of various diseases, such as esophageal cancer (Jammula et al., 2020), breast cancer (Zhang et al., 2020), asthma (Chen et al., 2021; Wang et al., 2022), etc.

In our study, we identified 20 pairs of CpG-gene combinations in the pollen season and 24 such combinations in the non-pollen season. Due to the property of allergen-specific excitation in allergic diseases, we believe that the non-pollen season gene results and shared genes reflect the common AR methylation changes, while the results of the pollen season reflect pollen-specific AR methylation changes. According to the WGCNA results, the methylation-driven genes of pollen and non-pollen seasons were distributed in different color modules (Supplementary Tables S3, S4), the genes within the modules have synergistic effects, indicating that there might be distinct mechanism for methylation regulation in the pollen season.

We have identified 4 shared methylation-driven genes for the pollen and non-pollen seasons, namely, *SP140*, *NRCAM*, *NAPRT*, and *FBP1*. Their functions are associated with allergic diseases both on allergic inflammation and oxidative stress levels. *SP140* is critical for transcriptional programs that uphold the macrophage state and also is a potentially novel gene contributing to IgE-dependent mast cells (MCs) activation (Fraschilla et al., 2022). *Fbp1* can aggravate oxidative stress-induced apoptosis by suppressing *Nrf2* signaling, which exerts a significant impact on the prevalence and severity of asthma (Hu et al., 2021).

Methylation-driven genes specific to the non-pollen season include *TNFRSF4*, *IL18R1*, *CCR5*, *SPRY1*, *LY86*, *SGK1*, *NFIL3*, *IL2RA*, *FRAT1*, *SPI1*, *SLC15A3* and *THRA* (Figure 4B). These genes are primarily discussed at the pathway level, their functional enrichment results involve signaling pathways related to cytokines and interleukins, which include cytokine-cytokine receptor interaction (hsa04060), cytokine-mediated signaling pathway (GO:0019221), and signaling by interleukins (R-HSA-449147) (Supplementary Figure S3D–F). The pathways involved in this process regulate the complex signaling of allergic inflammation by influencing the recruitment and dissipation of cytokines and interleukins, as well as downstream reactions. Our team’s previous research identified cytokine-cytokine receptor interaction as the key target pathway of Tuomin-Zhiti-Decoction for AR through proteomics and functional enrichment analysis subsequently (Cheng et al., 2022).

We concentrate on the methylation effects caused by pollen. Methylation-driven genes specific to the pollen season include *ZNF667-AS1*, *AHNAK*, *ORMDL3*, *RASSF1*, *TGFBR2*, *GTSF1* and *LEF1-AS1* (Figure 4A). Through literature retrieval, it has been confirmed that 4 out of the 7 unique genes are regulated by DNA methylation in their transcription (Gao et al., 2018; Walter et al., 2018; Wu et al., 2018; Ma et al., 2020), which may reflect the response of AR to pollen exposure. Based on data on olive pollen allergy (GSE54522), the first four of these genes (*ZNF667-AS1*, *AHNAK*, *ORMDL3* and *RASSF1*) were confirmed (Figure 5G). Previous studies have reported their association with allergic diseases, mainly involving the influence on genetic susceptibility and regulation of allergic inflammation. Research findings indicate that *AHNAK* triggers an inflammatory reaction through the activation of MCs (Song et al., 2023). Numerous genome-wide association studies have identified *ORMDL3* as a gene associated with asthma susceptibility (Moffatt et al., 2007; Galanter et al., 2008; James et al., 2019; Ntontsi et al., 2021). *ORMDL3* is a transmembrane protein found in the endoplasmic reticulum that regulates sphingolipid synthesis. The molecular mechanisms underlying *ORMDL3*’s pathologic functions in asthma are connected to its evolutionarily conserved role in the regulation of sphingolipid homeostasis (James et al., 2019). *TGFBR2* (Transforming Growth Factor Beta Receptor 2) is one of the major components of the transforming growth factor β (*TGFβ*) signaling (Weiss and Attisano, 2013). People with *TGF* receptor mutations are significantly more likely to develop allergic disorders (Frischmeyer-Guerrerio et al., 2013). At the pathway level, we have identified the *Hippo* signaling pathway (hsa04390) and the neutrophil degranulation-associated pathway (R-HSA-6798695). Recent research has proved that the *Hippo* signaling pathway can regulate the macrophage population size by mediating the microenvironment, which may be the basis for its association with AR (Zhou et al., 2022). Although neutrophils are not traditionally regarded as components of type 2 immunity, a growing number of studies confirm that neutrophils may contribute to the initiation of type 2 immune responses (Wark et al., 2002; Kämpe et al., 2011; Kämpe et al., 2012).

Motivated by the well-known asthma-related gene *ORMDL3*, we decided to investigate if the pollen season’s methylation effects could affect AR and contribute to the development of asthma. There is growing evidence showing a strong association between AR and asthma. Both conditions are epidemiologically and pathophysiologically related (Bergeron and Hamid, 2005; Compalati et al., 2010; Bousquet et al., 2019), and AR is considered an independent risk factor for the development of asthma (Compalati et al., 2010; Acevedo-Prado et al., 2022). The key disease targets for the progression of AR to asthma have yet to be elucidated. Therefore, we introduced external data (GSE101720) to explore DNA methylation as the underlying mechanism of the progression from AR to asthma. As seen in the above volcano plot (Figure 5A–D), with the disease proceeding from AR to asthma, there was a significant increase in the amount of DEGs in the airway mucosa, and the trend is reversed in the nasal mucosa, though the difference was not significant, indicating a transfer of the inflammatory site from the nasal mucosa to the airway mucosa.

We found the *ZNF667-AS1* gene was significantly downregulated within both the AR and AR + asthma groups in data GSE101720 (Figure 5E, F). Referring back to the modifications in the gene expression profile data GSE50101, we observed that *ZNF667-AS1* was also a downregulated expression DEG and that it also had the most significant change in the expression of the seven methylation-driven genes specific to the pollen season (Supplementary Table S3), leading us to conclude that it was the primary gene mediating the pollen effect. *ZNF667-AS1* is classified as a long-stranded non-coding RNA (lncRNA) with multifaceted activities. lncRNAs were proven to play an integral role in the pathogenesis of allergic disease by regulating the differentiation and apoptosis of hematopoietic stem cells, bone marrow cells, and the activation of monocytes, macrophages, and dendritic cells in immune regulation (Cheng et al., 2022). *ZNF667-AS1* has been the focus of recent research on inflammatory conditions and cancer with an emphasis on its function in the control of inflammatory pathways (Di Fiore et al., 2021; Zheng et al., 2021; Fan et al., 2022; Luan et al., 2022; Ma et al., 2022; Bohosova et al., 2023). Excitingly, we discovered that Liu et al.'s bioinformatics study (Liu et al., 2019), based on data GSE67472, identified *ZNF667-AS1* as an important gene involved in the pathogenesis of asthma, which is also downregulated in asthma. We applied WGCNA to investigate the function of *ZNF667-AS1* further, as the functional annotation of lncRNA is still in its exploratory stages. The blue module was the only significant module related to AR (*Cor* = 0.74, *p*-value < 0.05) (Figure 6D) and *ZNF667-AS1* was the only methylation-driven gene in the blue module. Excited by this outcome, we proceeded to perform pathway enrichment analysis on the genes associated with *ZNF667-AS1* in the blue module. The reason for doing so is that genes identified within the same module by WGCNA are assumed to have high synergistic changes. This characteristic has been frequently used in recent years for functional annotation of lncRNAs, specifically exploring the function of lncRNAs through annotating the functions of mRNAs co-expressed with them (Wang et al., 2023). What we discovered was that the most significant class of KOBAS-i pathway intelligent clustering results comprised 18 pathways, including *TLR* family, *Myd88*, *MAPK*, and oxidative stress. *ZNF667-AS1* may also be implicated in activating *TLR* and *MAPK* signaling pathways, according to gene set enrichment study conducted around the CpG site *cg05508084* corresponding to *ZNF667-AS1*. Pollen can directly interact with pattern recognition receptors (PRRs), particularly *TLR* family, as many studies have proved to date. This interaction can induce *TSLP* production and related type 2 inflammation, and it may also be related to reactive oxygen-mediated oxidative stress (Hosoki et al., 2015; Pointner et al., 2020; Pointner et al., 2021; Shumin et al., 2021; Guryanova et al., 2022). Based on our research, methylation of the CpG site *cg05508084* in response to pollen stimulation drives the low expression of *ZNF667-AS1*, which in turn triggers type 2 inflammation and oxidative stress by affecting the *Myd88*-dependent *TLR* family, which may ultimately promote the development of AR to asthma. *ZNF667-AS1* may serve as a crucial mediator and biomarker of allergic diseases.

As per the concept of the “unified airway”, the upper and lower respiratory tracts share common barrier and immune characteristics, making them a morphologically and functionally unified entity (Bachert et al., 2023; Wang et al., 2023), which can explain that allergy is not a disease of a specific organ, but a disorder of the whole respiratory tract (Compalati et al., 2010). This coincides with the concept of the allergic constitution, which is considered as the background and foundation for the development of allergic diseases by the theory of traditional Chinese medicine constitution (Wang et al., 2023). Our previous study (Sun et al., 2024) suggests that pathways such as T_H_1 and T_H_2 cell differentiation, *TLR* cascade are the key molecular characterization of allergic constitution. We believe that the occurrence of allergic disease is the result of a combination of innate constitution and environmental factors.

Dutch children and adolescents’ overall symptom scores are positively correlated with grass and birch pollen concentrations, according to a recent study (Tameeris et al., 2023). We hypothesize that methylation effects could be associated with symptoms throughout the pollen season. According to the WGCNA results, blue, brown, black, green, turquoise, and lightcyan modules show a strong correlation with the allergy symptom score (Figure 6D). Moreover, a number of researches have documented a connection between pollen and allergy symptoms (Tobías et al., 2003; Kiotseridis et al., 2013; Kmenta et al., 2016; Wang et al., 2018). Unfortunately, because we do not have pollen concentration information in our data sources, we cannot analyze whether it correlates with methylation levels. That is one of our study’s limitations. Our analysis method is mainly based on bioinformatics, the dependability of the results is obviously affected by the sample size and the depth of the sample data. Furthermore, the data we used was restricted to transcriptomics and epigenomics, further research is required at multiple omics levels, including proteomics and metabolomics. In order to uncover the long-term effects of pollen season on AR at a deeper level, it is intended that additional research can be conducted in the future.

## Conclusion

In this study, we identified the methylation-driven genes triggered by pollen and investigated their functions through bioinformatic analysis. Four of the seven key methylation-driven genes (*ZNF667-AS1*, *AHNAK*, *ORMDL3* and *RASSF1*) were validated by another olive pollen allergy data. By incorporating external data, we identified *ZNF667-AS1* as a crucial methylation-driven gene mediating pollen effects, which could be a key gene driving the conversion of AR to asthma. Its potential mechanism may involve the influence on *TLR* family, further mediating type 2 inflammatory responses and impacting the occurrence and development of AR and asthma. Our findings provide new insights into the interaction between AR and asthma, and pave the way for scientific research and development of therapeutic strategies. We hope there will be researches conducted *in vitro* and *in vivo* in the future to validate our findings.

## Data Availability

The original contributions presented in the study are included in the article/Supplementary Material, further inquiries can be directed to the corresponding authors.
