# Supplementary material for: Methylation-driven mechanisms of allergic rhinitis during pollen and non-pollen seasons using integrated bioinformatics analysis
Source: Front Genet. 2024 Apr 18;15:1242974. doi: 10.3389/fgene.2024.1242974 (PMC11063319; doi:10.3389/fgene.2024.1242974)
Supplement: Supplementary file 2 [file Image1.pdf]

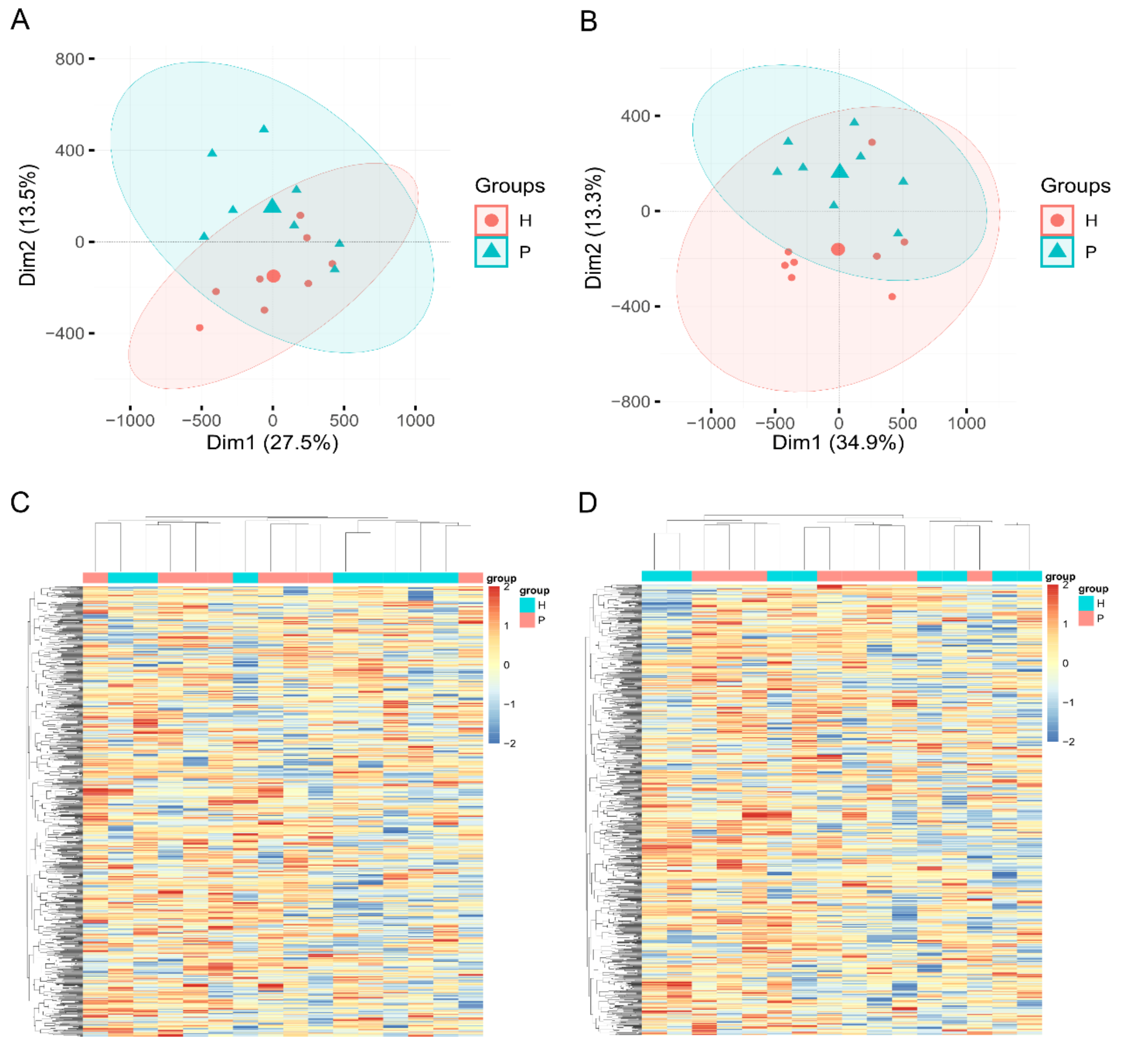


**FIGURE S1**

The principal component analysis (PCA) plot and heatmap of the methylation data (GSE50222). **(A)** PCA plot of samples during the pollen season. **(B)** PCA plot of samples during the non-pollen season. **(C)** Heatmap of sample during the pollen season. **(D)** Heatmap of samples during the non-pollen season.

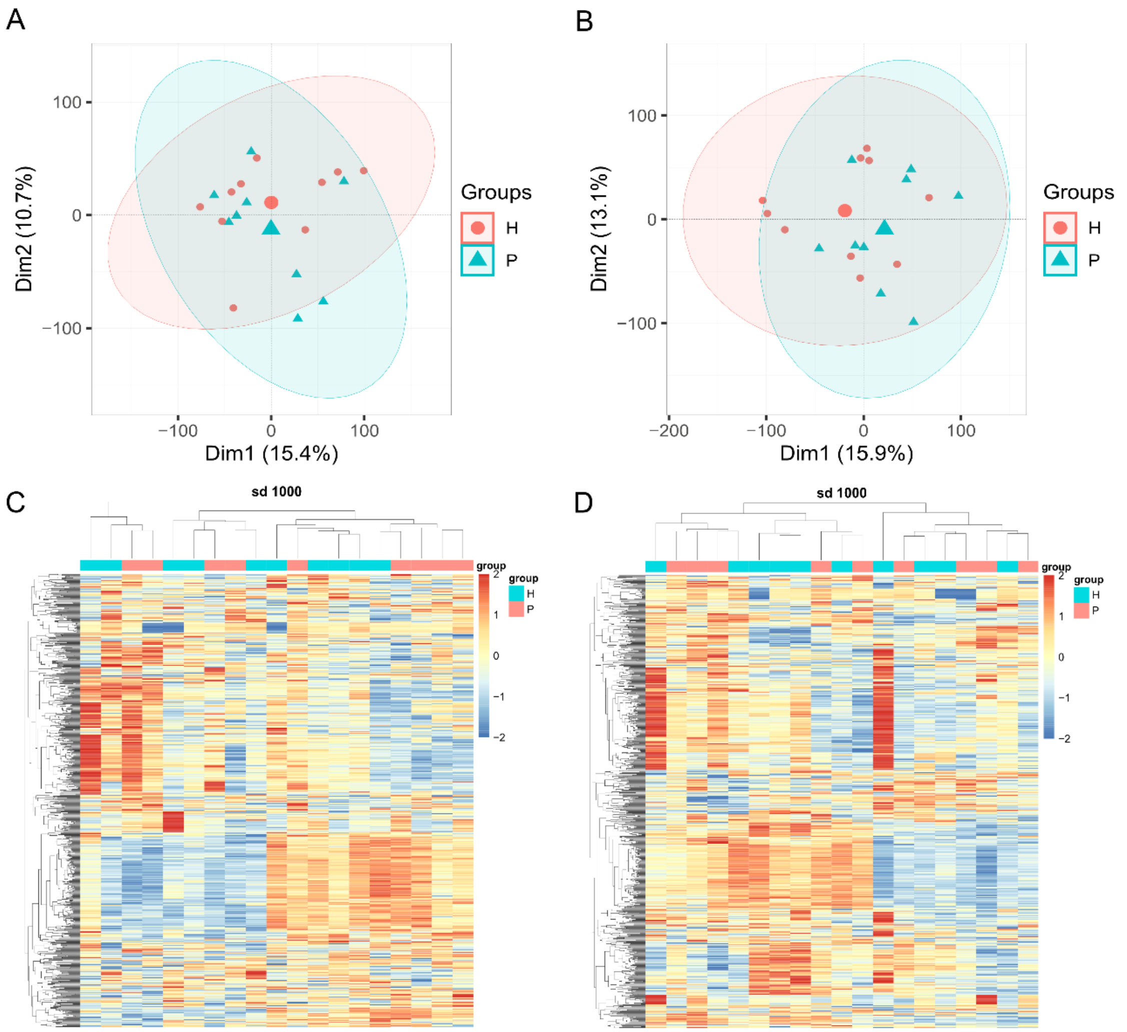


**FIGURE S2**

The principal component analysis (PCA) plot and heatmap of the gene expression data (GSE50101). **(A)** PCA plot of samples during the pollen season. **(B)** PCA plot of samples during the non-pollen season. **(C)** Heatmap of sample during the pollen season. **(D)** Heatmap of samples during the non-pollen season.

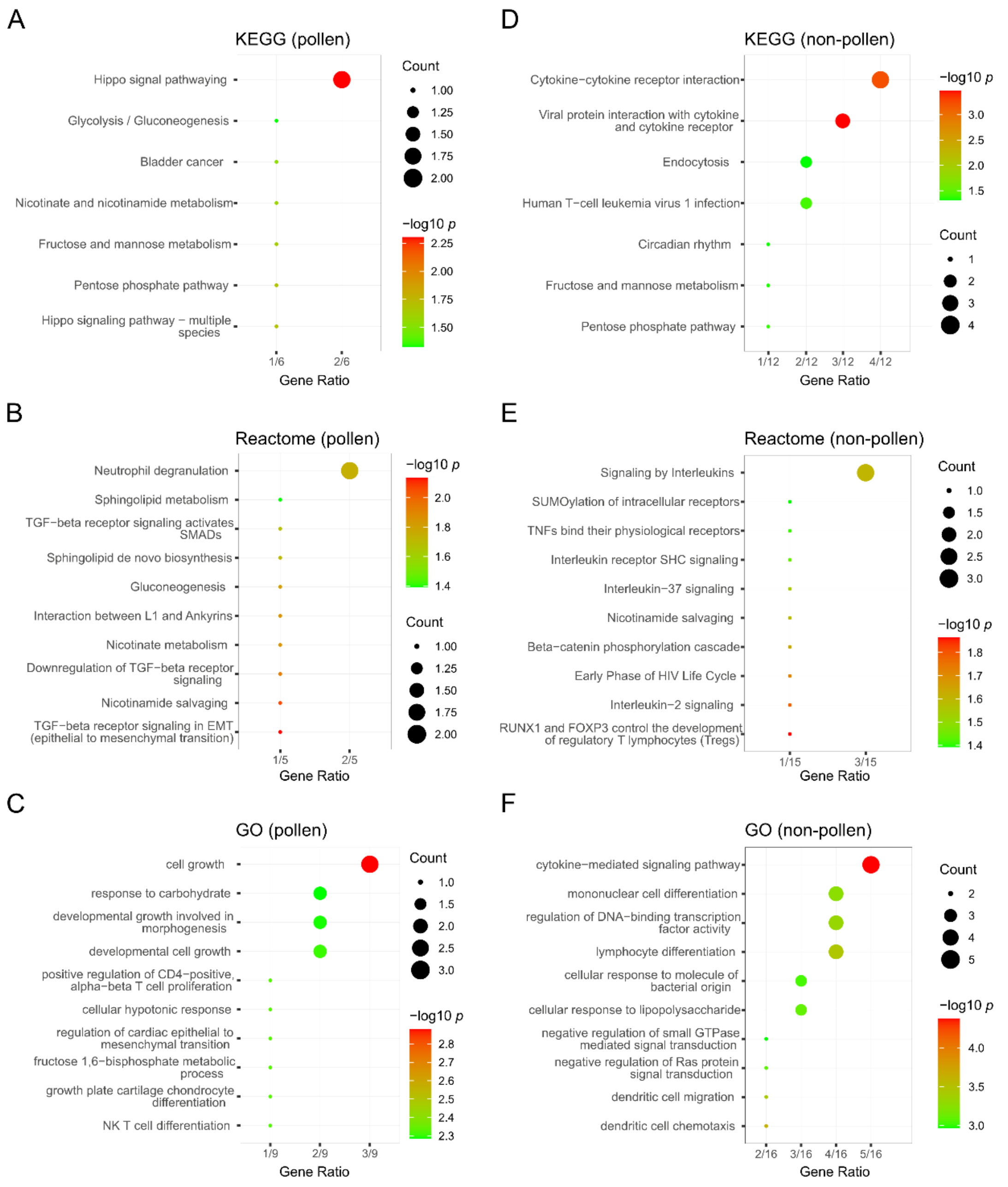


## FIGURE S3

Gene function enrichment analysis. **(A, D)** KEGG pathway map of methyiation-driven genes of the pollen season and the non-pollen season. **(B, E)** Reactome pathway map of methylation-driven genes of the pollen season and the non-pollen season. **(C, F)** Gene Ontology (GO) map of methylation-driven genes of the pollen season and the non-pollen season.

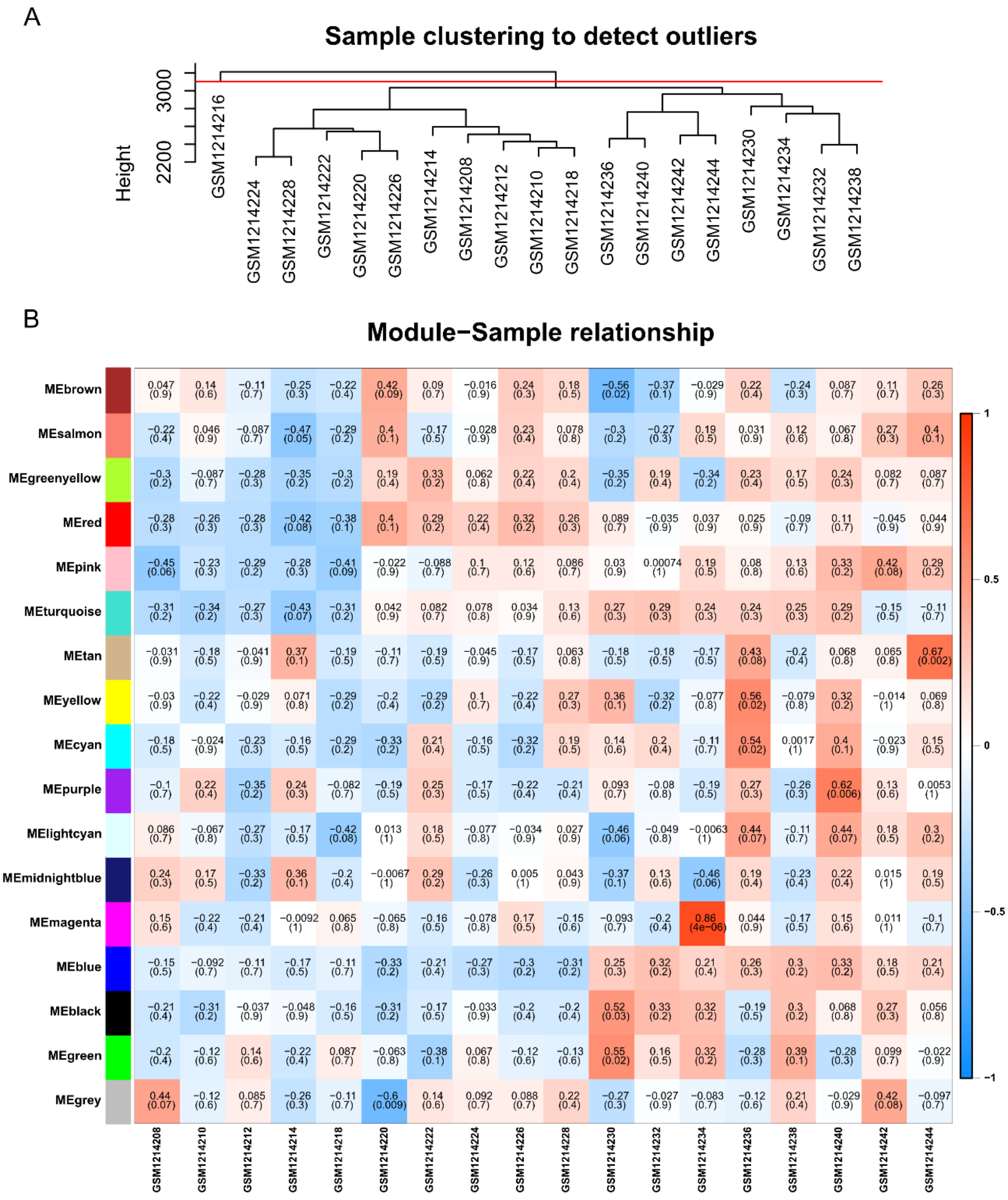


# FIGURE S4

Results plot of WGCNA. **(A)** Sample clustering plot of GSE50101. The height of cutoff line in red is 3100. **(B)** Correlation plot between modules and samples. WGCNA: weighted correlation network analysis.
